# Fecal Metabolomics for the Diagnosis of *Clostridioides difficile* Infection

**DOI:** 10.3390/diagnostics15182331

**Published:** 2025-09-15

**Authors:** Carlos Bea-Serrano, Andreu Belmonte-Domingo, Carolina Pinto-Pla, Ana Ferrer-Ribera, Sara Vela-Bernal, Ana Isabel de Gracia-León, Andrea de Castro-Oliver, Lucas Serna-Navarro, Celia Prades-Sirvent, David Ruiz-Raga, María José Galindo, María José Forner-Giner, María Rosa Oltra-Sempere

**Affiliations:** 1 Internal Medicine Department, Clinic University Hospital of Valencia, INCLIVA Biomedical Research Institute, 46010 València, Spain; 2 Department of Medicine, Faculty of Medicine and Dentistry, University of València, 46010 València, Spain

**Keywords:** *Clostridioides difficile*, fecal metabolomics, diagnosis, biomarkers, bile acids, SCFAs, amino acids

## Abstract

**Background:** *Clostridioides difficile* infection (CDI) is the leading cause of nosocomial diarrhea. Current diagnostic tools have difficulty distinguishing between colonization and active infection. This study evaluated the utility of fecal metabolomics in diagnosing CDI in hospitalized patients with acute diarrhea. **Methods**: We conducted a prospective observational study involving hospitalized adults with new-onset diarrhea during admission. Participants were stratified into groups based on clinical and microbiological findings: controls, *C. difficile* colonized and *C. difficile* infected. Fecal samples were analyzed using UPLC-MS/MS and GC-MS to quantify selected short-chain fatty acids, amino acids, and bile acids. Multivariate and univariate statistical analyses included PLS-DA, sPLSDA, and tests with FDR correction. **Results:** Infected patients exhibited significantly higher concentrations of SCFAs and notable alterations in bile acid profiles. Key discriminative metabolites included isovalerate, propionate, isobutyrate and alpha-aminobutyric acid. ROC curve analyses showed strong diagnostic performance for these markers, with AUC values exceeding 0.85. **Conclusions**: Fecal metabolomic profiling could effectively differentiate between colonization and infection in CDI among hospitalized patients with diarrhea. These results highlight the potential of metabolomic signatures to enhance the diagnostic precision for CDI.

## 1. Introduction

*Clostridioides difficile* infection (CDI) is the most common cause of healthcare-associated diarrhea in high-income countries and represents a growing public health challenge due to its increasing incidence, recurrence rates, and severity [1,2]. While diagnostic techniques have evolved—from enzyme immunoassays to nucleic acid amplification tests (NAATs)—the ability to distinguish between active infection and asymptomatic colonization remains limited [3]. This distinction is clinically critical, as up to 3–21% of hospitalized patients may be colonized by toxigenic *C. difficile* without presenting symptoms [4,5], and overtreatment based on NAATs alone can lead to unnecessary antibiotic exposure and costs. The concept of “colonization resistance” underscores the protective role of the gut microbiota against *C. difficile* overgrowth [6,7]. Antibiotic-induced dysbiosis disrupts this balance, allowing spore germination, vegetative proliferation, and toxin production. However, microbial composition alone may not capture the full picture. Increasing evidence suggests that the gut metabolome—the collective metabolic output of host and microbial activity—plays a decisive role in determining susceptibility to CDI [8].

Among the most relevant microbial metabolites are bile acids and short-chain fatty acids (SCFAs). Primary bile acids, such as cholic and chenodeoxycholic acid, can promote *C. difficile* germination, while secondary bile acids like deoxycholic and lithocholic acid inhibit its growth and toxin production [9,10]. CDI has been consistently associated with an increased ratio of primary to secondary bile acids, reflecting impaired microbial 7α-dehydroxylation capacity [11,12]. On the other hand, SCFAs—particularly butyrate, isobutyrate, and propionate—contribute to epithelial integrity and modulate immune responses. Their depletion has been linked to gut barrier dysfunction and may facilitate disease onset and progression, although previous studies in animal and human models have shown contradictory results [13,14,15]. Despite the growing body of evidence, few studies have directly compared the fecal metabolic signatures of patients with active CDI, asymptomatic colonization, and non-colonized controls [15,16,17]. This distinction is crucial, as it mirrors the real-world diagnostic dilemma clinicians face, particularly when microbiological results and clinical presentation are discordant.

In this study, we applied targeted fecal metabolomics to characterize and compare the metabolic profiles of hospitalized patients with confirmed CDI, toxigenic *C. difficile*-colonized patients, and non-colonized controls with acute onset diarrhea. We aimed to identify metabolite-level alterations, especially in bile acid and SCFA pathways, that could serve as discriminatory biomarkers for CDI. By focusing on clinically relevant patterns, our goal was to explore the potential of fecal metabolomics as a complementary tool in the diagnostic work-up of CDI.

## 2. Materials and Methods

### 2.1. Study Design

This was a single-center, experimental, prospective study conducted at a tertiary hospital between January 2019 and March 2023. Fecal samples were collected from hospitalized adult patients with new-onset diarrhea for whom *C. difficile* diagnostic testing was requested.

### 2.2. Study Population and Classification

Inclusion criteria were as follows: age ≥ 18, hospitalization for more than 48 h at inclusion, acute diarrhea during admission, and availability of *C. difficile* diagnostic results (PCR for toxin B and toxin B antigen immunochromatographic assay). Exclusion criteria were recent CDI treatment (<14 days) or incomplete microbiological data. Patients were classified into three groups: (1) Infection: PCR-positive and antigen-positive, meeting all clinical criteria for CDI (including diarrhea with ≥3 unformed stools in 24 h, fever or abdominal pain, risk factors for CDI, and absence of alternative causes); further subdivided into first episode (I) and recurrence (R); (2) Colonized (C): PCR-positive, antigen-negative, with diarrhea but not meeting full CDI clinical criteria; (3) Control (X): Negative for both PCR and toxin, but with acute diarrhea during hospitalization.

### 2.3. Ethics Statement

All data were pseudonymized. Data collection followed GDPR and Spanish biomedical research regulations. The study was approved by the INCLIVA Institutional Review Board (CEIm), reference number 2020/271, on 12 November 2020, and conducted in accordance with the Declaration of Helsinki (2013).

### 2.4. Sample Size

This was an exploratory translational study. Based on literature and feasibility, we selected 30 CDI patients (15 first-episode infections and 15 recurrent infections), 15 colonized patients, and 15 controls.

### 2.5. Clinical Data Collection

Demographic, clinical, microbiological, and therapeutic variables were extracted from electronic health records. Collected variables are detailed in Supplementary Table S1.

### 2.6. Sample Handling and Metabolomic Processing

Fecal metabolomic profiling was conducted using a combined analytical platform incorporating both liquid chromatography–tandem mass spectrometry (LC-MS/MS) and gas chromatography–mass spectrometry (GC-MS). A total of 77 metabolites were quantified, including bile acids, amino acids, and SCFAs. Detection rates exceeded 95% for nearly all SCFAs and amino acids. Quantitative normalization followed standard internal procedures appropriate for fecal metabolomics, and concentrations were corrected for wet fecal weight.

#### 2.6.1. Sample Preparation

Approximately 100–200 mg of each stool sample was aliquoted into 1.5 mL Eppendorf tubes and stored at −80 °C until analysis. Samples were thawed at room temperature, extracted in 600 µL CH_3_OH:PBS (1:1) using the PreCellys^®^ homogenizer, and centrifuged (12,000× *g*, 4 °C, 12 min). Supernatants were transferred to clean vials and processed according to the analytical platform described below.

#### 2.6.2. Bile Acids

Bile acids were quantified using a validated UHPLC-MS/MS protocol adapted from Ramos-García et al. [18]. Extracts were diluted with mobile phase (H_2_O:CH_3_CN 90:10, 0.1% HCOOH) and spiked with deuterated internal standards (2.1 µM each). Protein precipitation was achieved with cold methanol, followed by incubation (−20 °C, 20 min) and centrifugation (3500× *g*, 15 min, 4 °C). Methanol, propanol, acetic acid, ammonium acetate, acetonitrile, and formic acid were purchased from Merck Life Science S.L.U. (Madrid, Spain). Certified reference standards of bile acids (CA, CDCA, LCA, DCA, GCA, TCA, α-MCA, β-MCA, UDCA, HDCA, MCA, DHCA, HCA, GLCA, GUDCA, GHDCA, GCDCA, GDCA, GDHCA, GHCA, TLCA, TUDCA, THDCA, TCDCA, TDCA, TDHCA, THCA, and T-α-MCA) and deuterated internal standards (LCA-D4, CA-D4, GCDCA-D4, GCA-D4) were obtained from Steraloids (Newport, RI, USA). Sulfated bile acid standards and LCA-S-D4 were supplied by Qmx Laboratories Ltd. (Essex, UK). Analysis was performed on an ACQUITY UPLC system (Waters Ltd., Elstree, UK) coupled to a Xevo TQ-S triple quadrupole mass spectrometer (Waters, Manchester, UK) operated in negative electrospray ionization (ESI−) mode. Chromatographic separation was achieved using an ACQUITY BEH C8 column (100 × 2.1 mm, 1.7 µm; Waters) maintained at 60 °C. The mobile phases consisted of: A: Acetonitrile with 1 mM ammonium acetate (pH 4.15, adjusted with acetic acid), B: Acetonitrile:propanol (1:1, v/v). A gradient elution was applied as follows: 0–0.1 min, 10% B; 0.1–9.25 min, 10–35% B; 9.25–11.5 min, 35–85% B; 11.5–11.8 min, 85–100% B; 11.8–14.3 min, 100–10% B; and 14.3–16 min, 10% B. Injection volume: 5 µL. Detection was performed in MRM mode. Method validation followed FDA bioanalytical guidelines [19].

#### 2.6.3. Short-Chain Fatty Acids and Branched-Chain Amino Acids

SCFAs and branched-chain amino acids (BCAAs) were analyzed using a derivatization method based on propyl chloroformate, adapted from Ramos-Garcia et al. [20]. SCFAs, branched-chain amino acids (BCAAs), propyl chloroformate, pyridine, sodium hydroxide, n-hexane, and isotopically labeled internal standard caproic acid-D3 were obtained from Sigma-Aldrich (Madrid, Spain). The AccQTag Ultra derivatization kit for amino acids was purchased from Waters (Milford, MA, USA). Ultrapure water was prepared using the Q-POD^®^ system (Merck KGaA, Darmstadt, Germany). After derivatization, samples were analyzed using a GC 7890B system coupled to a 5977A quadrupole mass detector (Agilent Technologies, Santa Clara, CA, USA). Separation was achieved using a capillary HP-5MS column (30 m × 250 µm × 0.25 µm; Agilent J&W Scientific, Santa Clara, CA, USA).The oven program was: initial 50 °C (2 min), ramp to 70 °C at 10 °C/min, 85 °C at 3 °C/min, 110 °C at 5 °C/min, and finally 290 °C at 30 °C/min, holding for 8 min. Helium was used as the carrier gas at 1 mL/min. Data acquisition was performed in SIM mode using MassHunter B.07.01 software (Agilent, Santa Clara, CA, USA). Concentrations were normalized to wet stool weight. Analytical batches included quality control (QC) samples injected every 10 study samples, along with blanks and process controls. QC acceptance criterion: RSD < 25%. Method performance was validated for linearity, recovery, precision, and freeze–thaw stability.

#### 2.6.4. Amino Acids

Amino acids were derivatized using the AccQTag Ultra kit (Waters, Milford, MA, USA) following the manufacturer’s protocol. 10 µL of the fecal extract was mixed with 10 µL H_2_O and 40 µL isopropanol (0.1% v/v formic acid). Subsequently, 10 µL of the diluted sample was combined with 70 µL borate buffer and 20 µL of the AccQTag reagent (6-aminoquinolyl-N-hydroxysuccinimidyl carbamate). After vortexing, samples were incubated at 55 °C for 10 min and transferred to a 96-well plate for analysis. Amino acids were quantified using specific MRM transitions optimized for each analyte, combined with external calibration curves prepared from pure standards. System performance and quantification accuracy were monitored through QC injections at regular intervals. 

### 2.7. Metabolomics and Statistical Analysis

Statistical analysis of clinical variables was conducted using SPSS v29.0. Depending on the data type and distribution, group comparisons employed Student’s *t*-test, Mann–Whitney U test, chi-squared test, or Fisher’s exact test. Multivariate logistic regression was used to identify independent clinical predictors of CDI. Targeted metabolomics data were processed using MetaboAnalyst 6.0 [21] and MATLAB 9.14. To account for the high dimensionality and potential non-normal distribution of metabolite concentrations, non-parametric ANOVA and Mann–Whitney U tests were applied, with false discovery rate (FDR) correction to adjust for multiple comparisons. Key discriminatory metabolites were identified using Significance Analysis of Microarrays (SAM) (a feature selection method originally developed for high-dimensional data) based on delta thresholds and q-values. To evaluate the diagnostic separation between the CDI and control groups, dimensionality reduction and classification techniques were employed. Principal component analysis (PCA) was used for unsupervised pattern recognition. Partial least squares discriminant analysis (PLS-DA), a supervised multivariate technique, and its sparse variant (sPLS-DA) were applied to enhance group discrimination and pinpoint potential biomarker metabolites. Visualization tools such as clustered heatmaps and self-organizing maps (SOMs) provided intuitive representations of metabolic shifts associated with CDI. Missing values were conservatively imputed using the minimum detected positive value per metabolite, preserving biological relevance while minimizing distortion.

## 3. Results

### 3.1. Study Population and Sample Processing

**Figure 1:**
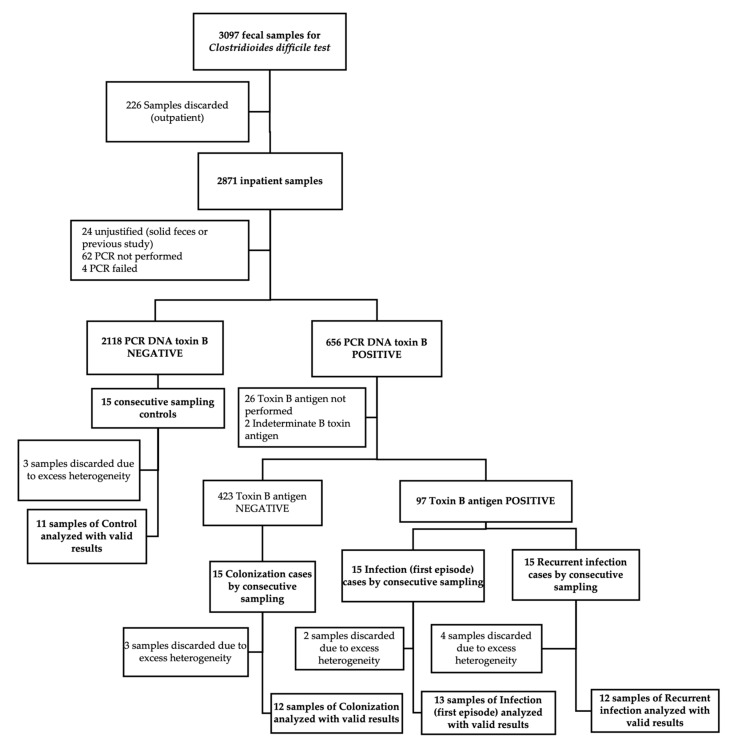
Study flowchart for sample selection and analysis.

A total of 60 fecal specimens were collected using a consecutive sampling strategy stratified by each of the four clinical groups defined by microbiological and clinical criteria: 30 individuals with confirmed CDI, subdivided into 15 with a primary episode (I) and 15 with a first documented recurrence (R); 15 individuals colonized with *C. difficile* (C); and 15 symptomatic control patients presenting with diarrhea but testing negative for the *C. difficile* toxin B gene by PCR (X). Each group was defined to explore distinct pathophysiological stages along the *C. difficile* infection spectrum. Following sample quality control, 12 fecal samples were excluded due to excess heterogeneity, defined by visual inspection (e.g., presence of undigested food or inconsistent stool texture), which could interfere with metabolomic profiling and mass spectrometry detection. This yielded a final analytical dataset of 48 fecal specimens (Figure 1). Mean concentrations for all analyzed metabolites are provided as supplementary material in Table S2.

**Figure 2:**
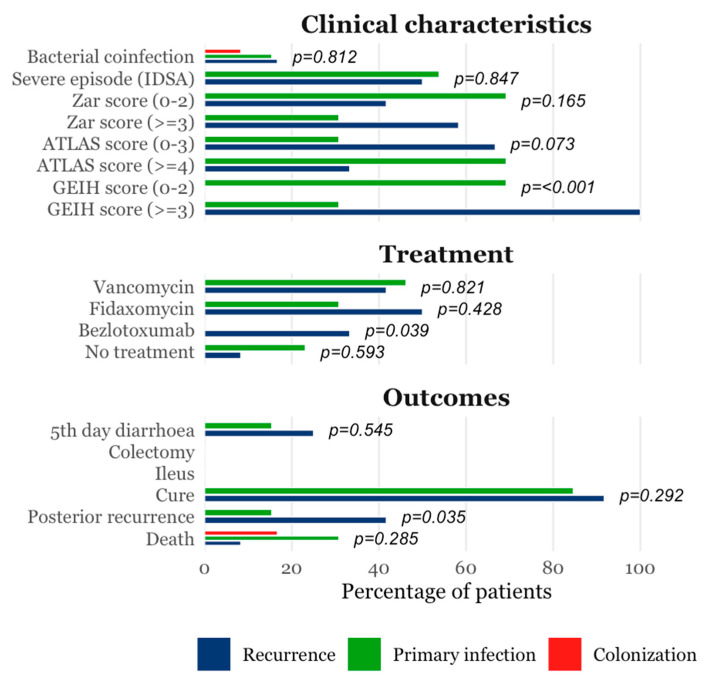
Clinical characteristics, treatment and evolution. GEIH, *Grupo Español Infección Hospitalaria*; IDSA, Infectious Disease Society of America.

Demographic profiles, including age and sex distribution, did not differ significantly across groups (Table 1). However, patients with active CDI exhibited a slightly higher burden of chronic comorbidities, notably heart failure (40% vs. 0% in colonized and 9% in controls, *p* = 0.003) and advanced-stage chronic kidney disease (32% in CDI vs. 0% in colonized vs. 18% in controls, *p* = 0.025). Antibiotic exposure was markedly elevated in the CDI group, with 80% of patients reporting more than 10 cumulative days of antibiotic therapy in the preceding month (vs. 8.3% in colonized and 18.2% in controls, *p* < 0.001). While these significant clinical differences exist, subsequent analysis indicates they were not the primary drivers of the observed metabolomic signature. Other epidemiological characteristics, risk factors for CDI and clinical characteristics are summarized in Table 1 and Table 2 and Figure 2. Severity parameters and microbiological diagnostics tests results (including Ct values for PCR) among different groups at diagnosis are presented in Table A1 and Table A2, respectively.

### 3.2. Global Metabolomic Trends

**Figure 3:**
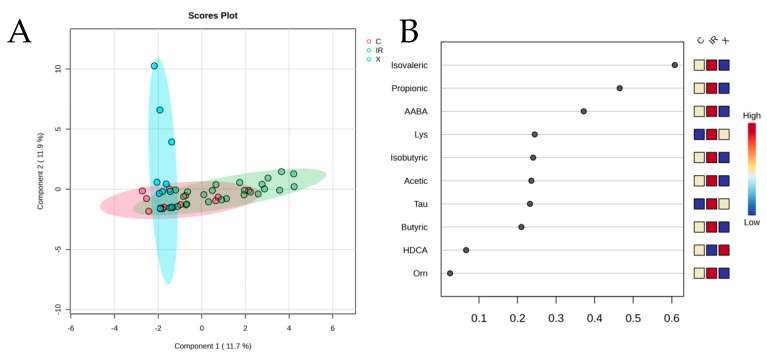
PLS-DA with three groups: CDI (IR), *C. difficile*-colonized (C), and controls (X). (**A**) Scores plot showing separation of fecal metabolomic profiles among patients with CDI (IR, green), colonized (C, red), and non-colonized controls (X, blue). Each dot represents an individual sample, and ellipses indicate the 95% confidence interval for each group. (**B**) Variable loadings plot corresponding to Component 1 of the PLS-DA model, highlighting the top metabolites contributing to class separation. Metabolites such as isovalerate, propionate, and AABA showed high discriminative power.

PCA revealed partial segregation between groups, with controls showing the most distinct separation from CDI patients. The first two principal components captured approximately 31% of the total variance. SCFAs contributed prominently to PC1, though their levels were notably elevated in *C. difficile*-positive samples. PLS-DA supported this trend, showing clear separation between controls and *C. difficile*-positive patients, with substantial overlap between colonized and infected groups. These data suggest that *C. difficile* colonization—irrespective of symptomatology—is also associated with a metabolically distinct fecal profile (Figure 3).

### 3.3. Differential Metabolites and Diagnostic Stratification

**Figure 4:**
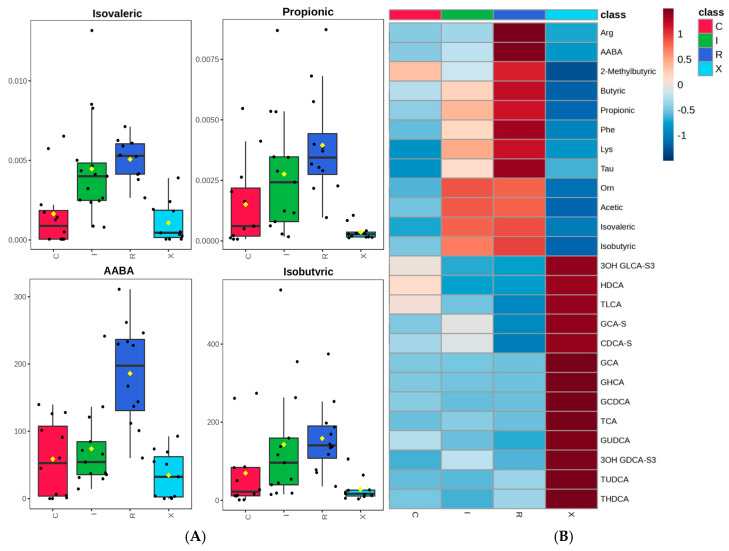
(**A**) Boxplots showing the distribution of isovaleric acid, propionic acid, AABA, and isobutyric acid concentrations across study groups. These metabolites exhibited the most marked differences between groups, with higher levels generally observed in infection states, particularly recurrent cases. (**B**) Heatmap of selected fecal metabolites across the four study groups. Metabolite intensities were autoscaled prior to visualization. The panel highlights distinct group-level differences, including elevated levels of SCFAs and amino acid derivatives in infected patients (I+R), and reduced concentrations of secondary bile acids, particularly in recurrent CDI (R). Rows represent metabolites and columns the mean concentration for each group; the color scale denotes relative abundance (red: higher; blue: lower).

Several metabolites, particularly SCFAs and select amino acids, were significantly elevated in *C. difficile*-positive patients compared to controls. Propionate, isovalerate, and alpha-aminobutyric acid (AABA) showed the most consistent increases in infected patients. Boxplots and heatmaps revealed that these metabolites were markedly enriched in infection states, particularly in cases with recurrence (Figure 4).

When comparing active CDI (first episode + recurrent infection) to non-infected patients (colonized + controls), isovalerate, propionate, and AABA were also significantly higher in the CDI group (*p* < 0.001; FDR < 0.01). Hierarchical clustering using these top metabolites provided good separation of infected from non-infected samples. Notably, SCFA elevation in CDI was observed in a much smaller proportion of colonized individuals, reinforcing its link with active infection.

**Figure 5:**
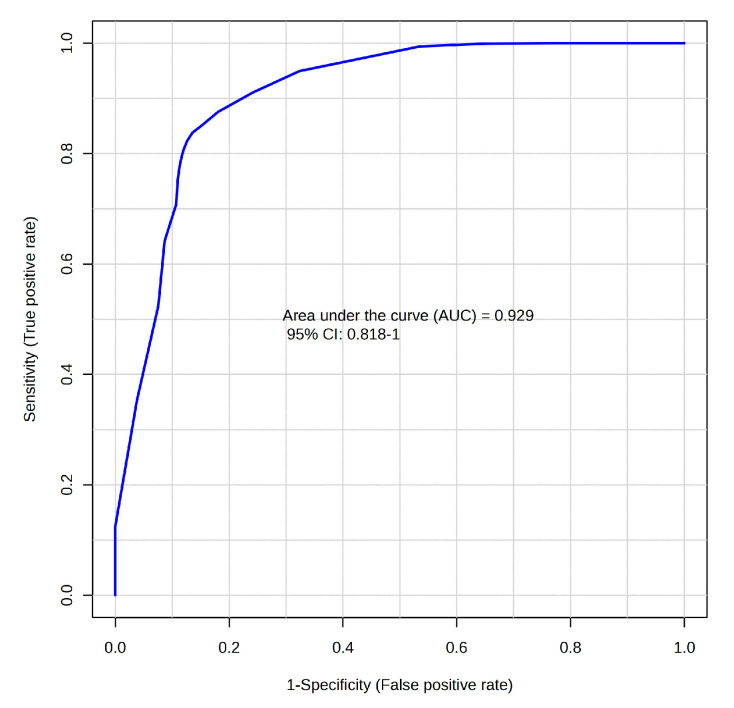
ROC curve showing the diagnostic performance of the AAA/propionate ratio for distinguishing CDI from non-infected individuals (colonized and controls). The analysis was performed using MCCV with 100 iterations.

Receiver operating characteristic (ROC) analysis confirmed the diagnostic potential of these analytes. Isovalerate and propionate achieved AUCs of 0.892 (0.786–0.979) and 0.849 (0.731–0.947) for identifying CDI, respectively. Metabolite ratios improved discriminative capacity further: aminoadipic acid (AAA)/propionate yielded an AUC of 0.929 (0.818–1.000) in 100-fold MCCV (Figure 5). Restricting analysis to CDI versus strictly non-colonized controls resulted in even higher diagnostic precision. Isovalerate alone produced an AUC of 0.941 (0.875–1.000). The AUC, sensitivity, and specificity of the main metabolites and ratios with biomarker potential for identifying CDI were determined through logistic regression with 10-fold cross-validation (Table 3).

### 3.4. Recurrent Infection and Group-Specific Trends

The overall recurrence rate in our cohort was 28%, comparable to rates reported in previous studies (Seekatz et al., 34% [22]; Dawkins et al., 29% [23]; Khanna et al., 28.5% [24]) and higher than the 15% reported by Guh et al. [25]. Notably, none of our patients received metronidazole, which has been associated with increased recurrence risk; all were treated with vancomycin or fidaxomicin. When comparing recurrent CDI cases (R) to primary infections (I), modest differences in fecal metabolite concentrations were observed. Recurrent cases tended to exhibit higher levels of AABA and select SCFAs such as isovalerate and propionate. However, only AABA consistently reached statistical significance in the Mann–Whitney U test (FDR 0.045). To further explore this observation, we examined the metabolomic profiles of individuals who experienced a recurrence after their sample had been collected during the primary episode. Individuals in their first infection episode clustered more closely with the recurrent infection group in both unsupervised and supervised analyses, reinforcing the potential of fecal metabolomics to capture features associated with recurrence risk even prior to clinical relapse.

### 3.5. Influence of Comorbidities and Risk Factors

**Figure A1:**
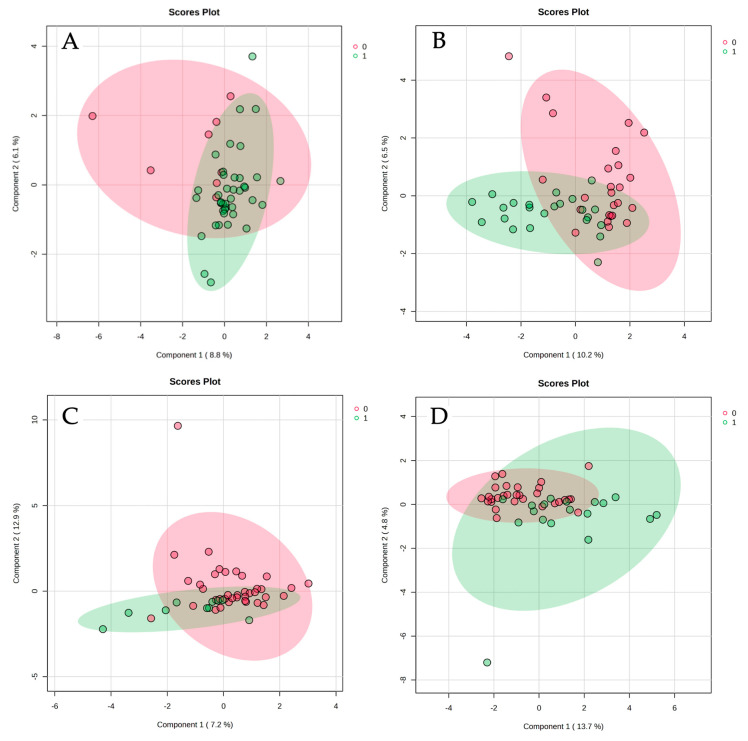
sPLS-DA of the fecal metabolomic profile according to the presence or absence of selected comorbidities and CDI risk factors that showed differences in their distribution between groups. (**A**) Previous antibiotic exposure; (**B**) antibiotic treatment longer than 10 days; (**C**) diabetes mellitus; (**D**) heart failure.

To assess potential confounding effects, we analyzed the fecal metabolomic profiles stratified by the presence or absence of major comorbidities and known risk factors for CDI, including heart failure, advanced chronic kidney disease, and recent antibiotic use. No significant differences in metabolite abundance or composition were observed between patients with and without these risk factors (Figure A1). This suggests that the metabolic alterations identified are primarily associated with infection status rather than underlying host factors, strengthening the specificity of the metabolomic signature for CDI.

## 4. Discussion

This study demonstrates that targeted fecal metabolomics can effectively differentiate between CDI, colonization, and non-infectious diarrhea in hospitalized patients. Elevated levels of specific SCFAs—notably isovalerate, propionate, and isobutyrate—along with AABA, emerged as robust metabolic signatures of active CDI. Diagnostic accuracy improved further through the construction of metabolite ratios, such as AAA/SCFAs and GCDCA/SCFAs, achieving AUC values exceeding 0.85. AAA was included in several metabolite ratios that achieved high AUC values, given its relatively stable concentrations across all study groups, providing a consistent reference point for comparative analysis. These results highlight the potential of metabolomic signatures to differentiate not only between infected and control individuals but also to distinguish infection from colonization—an enduring diagnostic challenge.

These findings are consistent with and extend previous research, particularly the landmark study by Robinson et al. [15], which also identified 4-methylpentanoic acid (4-MPA), an SCFA product of Stickland fermentation, an anaerobic amino acid fermentation pathway used by certain gut bacteria, as a potential CDI biomarker. More recently, a large multi-omics study of hospital-acquired diarrhea by Bosnjak et al. [26] likewise reported elevated levels of several Stickland by-products in CDI, including 4-MPA, isovalerate, and isobutyrate, further corroborating our observations. Unlike these cohorts, our study incorporated a more granular clinical characterization and stratification into first infection, recurrence, colonization, and control groups. The observed enrichment of SCFAs in infected patients aligns with the anaerobic fermentative metabolism of *C. difficile*, particularly via the Stickland reaction. This contrasts with prior assumptions that SCFA depletion is a hallmark of dysbiosis and, therefore, was associated with CDI [13,27]. Instead, our data suggest that certain SCFAs may accumulate specifically in the setting of *C. difficile* overgrowth, likely due to bacterial metabolic activity rather than host or commensal dysfunction. Notably, in our study, isovalerate exhibited excellent discriminatory capacity, reinforcing the role of leucine fermentation pathways in CDI pathogenesis. In addition to SCFAs, AABA showed strong discriminatory performance, especially in recurrent infections. While previously underappreciated, AABA is a non-proteinogenic amino acid linked to nitrogen export during bacterial amino acid metabolism [28] and may reflect intensified metabolic flux in recurrent disease. This supports its potential role as a recurrence-specific biomarker.

The profile of bile acids also revealed group-specific patterns. As expected from prior studies, infected patients showed relatively lower levels of secondary bile acids, consistent with a loss of microbial dehydroxylation activity [12,29]. Though these differences were less pronounced than those observed for SCFAs, ratios involving GCDCA and secondary bile acids added diagnostic value, particularly when distinguishing between infection and colonization. This is in line with experimental data showing that primary bile acids promote *C. difficile* germination, while secondary bile acids inhibit its vegetative growth and toxin activity [10,30].

It is worth noting that in our exploratory PCA, the first two components explained approximately 31% of the total variance. While this percentage may seem modest, it is consistent with the inherent complexity of fecal metabolomic datasets, which capture highly multidimensional information influenced by numerous host and microbial factors. Despite this, both PCA and PLS-DA revealed clear separation between controls and *C. difficile*-positive patients, supporting the biological relevance of the metabolomic differences observed.

Importantly, we found no significant influence of major comorbidities (e.g., DM, heart failure), antibiotic duration, or common medications (e.g., PPIs, antidepressants) on metabolomic patterns within clinical groups. Although the use of oral nutritional supplements (ONS) was more frequent in controls—possibly contributing to diarrhea in some of these patients—it did not significantly affect fecal metabolomic profiles. This supports the hypothesis that the metabolic shifts observed are primarily attributable to infection status, enhancing their potential utility as specific diagnostic tools.

While not designed for prediction, exploratory analyses suggested that metabolomic fingerprints may offer early indications of recurrence risk. In particular, samples from patients who later relapsed showed a profile more similar to recurrent cases at baseline. This echoes observations from Dawkins et al. [23], who highlighted metabolomic delays in post-treatment microbiome recovery as potential predictors of recurrence.

Some experts have proposed that *C. difficile* colonization and infection should be understood as a pathophysiological continuum rather than as two distinct clinical entities [31]. This spectrum-based model considers the interplay of multiple host and microbial factors that modulate disease progression. In our study, the proximity of metabolomic profiles observed between some colonized individuals and patients with active infection supports this concept and suggests that a subset of colonized patients may represent a preclinical stage of CDI. Identifying such high-risk individuals early could have important preventive implications, although the central clinical challenge remains determining when targeted treatment is truly warranted.

Although the comprehensive targeted fecal metabolomics approach used in this study relies on LC-MS/MS and GC-MS platforms that are currently confined to research settings, our findings highlight the translational potential of specific metabolites as diagnostic biomarkers. By improving the ability to distinguish between C. difficile colonization and true infection, these biomarkers could complement NAATs, which have high sensitivity but limited specificity and may lead to overtreatment. In the future, once validated in larger multicenter cohorts, simplified assays quantifying a small set of key metabolites directly from stool samples could enable practical implementation in clinical laboratories without requiring complex mass spectrometry workflows.

A distinguishing feature of our work is the comprehensive and systematic assessment of clinical characteristics and potential risk factors, which was conducted in far greater detail than in any previous metabolomic study of *C. difficile* infection, thereby allowing a more robust adjustment for possible confounders. Another strength of this study is the rigorous methodology applied in fecal sampling, storage, and analysis. Sample handling was tightly standardized to minimize variability related to collection, homogenization, and freeze–thaw cycles—critical issues in fecal metabolomics. Dual-platform analysis using GC-MS and UPLC-MS/MS enabled broad coverage of both volatile and non-volatile compounds, ensuring reliable quantification of key metabolite classes. 

Despite these promising findings, several limitations should be noted. First, the study was exploratory, with a relatively small sample size and a cross-sectional design, which limit the statistical power, subgroup analyses, and the generalizability of our results. Consequently, the associations observed between specific metabolites and infection status should be interpreted with caution and considered hypothesis-generating. Although formal power calculations were not feasible due to the lack of prior effect size estimates for most metabolites, we acknowledge the potential for type I and type II errors as well as model overfitting. To mitigate these risks, we employed repeated cross-validation and multiple testing correction strategies; nevertheless, independent validation in larger, multicenter prospective studies is required to confirm the diagnostic potential of the identified biomarkers. Second, although all patients received standardized hospital diets and detailed information on antibiotic and other relevant drug use was collected, residual confounding from unmeasured dietary variability and concomitant medications cannot be ruled out. Given the well-known impact of both factors on the fecal metabolome, the lack of full control over these variables represents a significant limitation of our study and should be considered when interpreting our findings. Third, the lack of concurrent microbiome sequencing limits our ability to link metabolic shifts with specific microbial taxa. Future work integrating metagenomics with metabolomics will be critical to clarify host–microbiota–metabolite interactions.

## 5. Conclusions

This study adds to the growing evidence that fecal metabolomics may help refine the diagnostic approach to CDI. Our findings suggest that SCFAs, AABA, and bile acid ratios could complement existing microbiological testing by improving specificity—particularly in distinguishing colonization from infection, a current clinical challenge. These metabolites may therefore represent the basis for the development of adjunct diagnostic tools, although this hypothesis requires rigorous validation. Prospective studies in larger, multicenter cohorts with longitudinal follow-up and integration of microbiome sequencing will be essential to confirm these associations and assess their clinical utility. Ultimately, if validated, the incorporation of metabolomic biomarkers into clinical workflows has the potential to enable earlier and more accurate diagnoses, optimize treatment decisions, and reduce unnecessary antibiotic use.

## Figures and Tables

**Table 1:** Epidemiological characteristics and comorbidities.

		Infection (*n* = 25)	Colonization (*n* = 12)	Control (*n* = 11)	*p*-Value
Female sex		13 (52%)	7 (58.3%)	3 (27.3%)	0.266
Neoplasia	Located	3 (12.0%)	0 (0.0%)	1 (9.1%)	0.560
	Metastatic	6 (24.0%)	4 (33.3%)	2 (18.2%)	
Leukemia		1 (4.0%)	2 (16.7%)	1 (9.1%)	0.444
Lymphoma		2 (8.0%)	2 (16.7%)	1 (9.1%)	0.732
Liver	Mild to moderate	2 (8.0%)	2 (16.7%)	0 (0.0%)	0.392
	Severe/cirrhosis	1 (4.0%)	0 (0.0%)	0 (0.0%)	
Moderate–severe CKD		8 (32.0%)	0 (0.0%)	2 (18.2%)	0.025
Hemodialysis		1 (4.0%)	0 (0.0%)	1 (9.1%)	0.466
IBD		1 (4.0%)	0 (0.0%)	2 (18.2%)	0.164
Diabetes mellitus	No TO damage	1 (4.0%)	1 (8.3%)	4 (36.4%)	0.024
	With TO damage	4 (16.0%)	0 (0.0%)	0 (0.0%)	
Dementia		2 (8.0%)	1 (8.3%)	2 (18.2%)	0.662
CVD		1 (4.0%)	1 (8.3%)	1 (9.1%)	0.794
Heart failure		10 (40.0%)	0 (0.0%)	1 (9.1%)	0.003
Ischemic heart disease		3 (12.0%)	0 (0.0%)	1 (9.1%)	0.288
Peripheral artery disease		1 (4.0%)	0 (0.0%)	1 (9.1%)	0.466
COPD		1 (4.0%)	1 (8.3%)	0 (0.0%)	0.510
Connective tissue disease		1 (4.0%)	2 (16.7%)	0 (0.0%)	0.199
Peptic ulcer		2 (8.0%)	1 (8.3%)	1 (9.1%)	0.994
Immunocompromised		9 (36.0%)	5 (41.7%)	5 (45.5%)	0.854
Charlson	≤5	11 (44.0%)	6 (50.0%)	7 (63.6%)	0.552
	6 o+	14 (56.0%)	6 (50.0%)	5 (36.4%)	

CKD, chronic kidney disease; COPD, chronic obstructive pulmonary disease; CVD, cerebrovascular disease; IBD, inflammatory bowel disease; TO, target organ.

**Table 2:** Risk factors for CDI among patients included in the study.

Risk Factor		Infection (*n* = 25)	Colonization (*n* = 12)	Control (*n* = 11)	*p*-Value
Previous ATB use		25 (100%)	9 (75.0%)	7 (63.6%)	0.009
Number of previous ATB	0–2	14 (56.0%)	9 (75.0%)	6 (54.5%)	0.435
	>2	11 (44.0%)	3 (25.0%)	5 (45.5%)	0.489
Type of previous ATB	Quinolones	12 (48.0%)	4 (33.3%)	4 (36.4%)	0.641
	Lincosamides	1 (4.0%)	1 (8.3%)	0 (0.0%)	0.606
	Cephalosporins	20 (80.0%)	6 (50.0%)	5 (45.5%)	0.065
	Penicillins	12 (48.0%)	3 (25.0%)	1 (9.1%)	0.058
	Carbapenems	2 (8.0%)	2 (16.7%)	5 (45.5%)	0.029
	Oxazolidinones	3 (12.0%)	0 (0.0%)	4 (36.4%)	0.041
Duration of previous ATB	≤10 days	5 (20.0%)	11 (91.7%)	9 (81.8%)	<0.001
	>10 days	20 (80.0%)	1 (8.3%)	2 (18.2%)	—
Concomitant ATB use		11 (44.0%)	5 (41.7%)	5 (45.5%)	0.983
ONS		2 (8.0%)	1 (8.3%)	5 (45.5%)	0.026
Antidepressants		10 (40.0%)	7 (58.3%)	1 (9.1%)	0.032
PPI/AntiH2		21 (84.0%)	8 (66.7%)	8 (72.7%)	0.467

AntiH2, H2 antihistamines; ATB, antibiotic; ONS, oral nutritional supplement; PPI, proton pump inhibitors; WBC, white blood cells.

**Table 3:** Main metabolites and ratios with the highest potential as biomarkers for CDI and for recurrent infection, identified through logistic regression with 10-fold cross-validation.

Metabolite/Ratio	Condition	AUC	Sensitivity	Specificity
AAA/propionate	CDI (I+R vs. X+C)	0.883 (0.785–0.982)	0.783 (0.783–0.951)	0.880 (0.753–1.000)
AAA/isovalerate	CDI (I+R vs. X+C)	0.893 (0.776–1.000)	0.880 (0.880–1.000)	0.833 (0.622–1.000)
GCDCA/isobutyrate	CDI (I+R vs. X)	0.942 (0.868–1.000)	0.995 (0.950–1.000)	0.880 (0.753–1.000)
AABA	Recurrent infection (R vs. I)	0.888 (0.700–1.000)	0.917 (0.917–1.000)	0.769 (0.540–0.998)

AAA, aminoadipic acid; AABA, alpha-aminobutyric acid; GCDCA, glycochenodeoxycholic acid.

## Data Availability

The data presented in this study are available on request from the corresponding author due to ethical and privacy restrictions. The dataset contains individual-level patient information for which specific consent for public data sharing was not obtained.

## Supplementary Materials

The following supporting information can be downloaded at: https://www.mdpi.com/article/10.3390/diagnostics15182331/s1, Table S1: Demographic, epidemiological, and clinical variables; Table S2: Concentrations of metabolites in stool samples.

## Abbreviations

The following abbreviations are used in this manuscript:

AAA	Aminoadipic acid
AABA	Alpha-aminobutyric acid
ANOVA	Analysis of variance
BEH	Ethylene-bridged hybrid (chromatography column technology)
CDI	*Clostridioides difficile* infection
CKD	Chronic kidney disease
COPD	Chronic obstructive pulmonary disease
CVD	Cardiovascular disease
DA	Discriminant analysis
DM	Diabetes mellitus
ESI	Electrospray ionization
FDA	Food and Drug Administration
FDR	False discovery rate
GC-MS	Gas chromatography–mass spectrometry
GCDCA	Glycochenodeoxycholic acid
GDPR	General Data Protection Regulation
GEIH	*Grupo de Estudio de Infección Hospitalaria* (Spanish Hospital Infection Study Group)
IBD	Inflammatory bowel disease
IDSA	Infectious Diseases Society of America
LC-MS	Liquid chromatography–mass spectrometry
MCCV	Monte Carlo cross-validation
MPA	Methylpentanoic acid
MRM	Multiple reaction monitoring
MS	Mass spectrometry
NAAT	Nucleic acid amplification tests
PBS	Phosphate-buffered saline
PCA	Principal component analysis
PCR	Polymerase chain reaction
PLS-DA	Partial least squares–discriminant analysis
PPI	Proton pump inhibitors

## Appendix A

**Table A1:** Severity parameters among different groups at diagnosis.

	Recurrence	Infection	Colonization	Control	*p*-Value
Temperature (°C)	36.6 (IQR 36.3–37.8)	36.2 (IQR 36–37.4)	36.3 (IQR 36.0–36.8)	36.0 (IQR 36.0–38.0)	0.544
WBC (cels/μL)	14,015 (IQR 7080–17,562)	11,000 (IQR 7265–24,970)	7765 (IQR 2630–9520)	7170 (IQR 5660–10,080	0.069
Albumin (g/dL)	3.2 (±0.6)	3.03 (±0.7)	3.39 (±0.7)	2.9 (±0.7)	0.280
Creatinine (mg/dL)	0.79 (IQR 0.59–1.14)	1.60 (IQR 0.61–2.37)	0.96 (IQR 0.59–1.26)	0.83 (IQR 0.54–1.46)	0.586

WBC, white blood cells.

**Table A2:** Microbiological diagnostic tests of CDI in the included patients.

Test		Infection	Colonization	Control
Ct PCR DNAtoxB	≤25	22 (88.0%)	2 (16.7%)	–
	>25	3 (12.0%)	10 (83.4%)	–
GDH antigen	Positive	21 (84.0%)	5 (41.7%)	0 (0.0%)
	Negative	0 (0.0%)	5 (41.7%)	10 (90.9%)
	Not performed	4 (16.0%)	2 (16.7%)	1 (9.1%)
Toxin B IC	Positive	25 (100%)	0 (0.0%)	0 (0.0%)
	Negative	0 (0.0%)	12 (100%)	11 (100%)

Ct, cycle threshold; GDH, glutamate dehydrogenase; IC, immunochromatographic test.
