# Supplementary material for: Fecal Metabolomics for the Diagnosis of Clostridioides difficile Infection
Source: Diagnostics (Basel). 2025 Sep 15;15(18):2331. doi: 10.3390/diagnostics15182331 (PMC12468941; doi:10.3390/diagnostics15182331)
Supplement: Supplementary file 1 [file diagnostics-15-02331-s001.zip › diagnostics-3852017-supplementary.pdf]

Table S1. Demographic, epidemiological and clinical variables

Variable	Type
Sex	Qualitative, nominal
Age	Quantitative, continuous
Tobacco use	Qualitative, nominal
Alcohol use	Qualitative, nominal
Use of other toxic substances	Qualitative, nominal
History of active solid organ neoplasm	Qualitative, nominal
History of hematologic malignancy	Qualitative, nominal
History of immunosuppression	Qualitative, nominal
History of HIV/AIDS infection	Qualitative, nominal
History of arterial hypertension	Qualitative, nominal
History of dyslipidemia	Qualitative, nominal
History of diabetes mellitus	Qualitative, nominal
History of obesity	Qualitative, nominal
History of chronic kidney disease and/or hemodialysis	Qualitative, nominal
History of ischemic heart disease	Qualitative, nominal
History of acute myocardial infarction	Qualitative, nominal
History of chronic heart failure	Qualitative, nominal
History of peripheral arterial disease	Qualitative, nominal
History of COPD	Qualitative, nominal
History of other respiratory diseases	Qualitative, nominal
History of liver disease	Qualitative, nominal
History of gastric ulcer	Qualitative, nominal
History of dementia	Qualitative, nominal
History of cerebrovascular accident	Qualitative, nominal
History of other chronic neurological diseases	Qualitative, nominal
History of connective tissue disease	Qualitative, nominal
History of chronic inflammatory bowel disease	Qualitative, nominal
Other comorbidities	Qualitative, nominal
Charlson comorbidity index	Quantitative, discrete
History of *Clostridioides difficile* infection	Qualitative, nominal
Concomitant bacterial infection	Qualitative, nominal
Concomitant antibiotic use	Qualitative, nominal
Previous antibiotic exposure	Qualitative, nominal
Number of previous antibiotics	Quantitative, discrete
Duration of previous antibiotic therapy	Quantitative, continuous
Previous treatment with quinolones	Qualitative, nominal
Previous treatment with lincosamides	Qualitative, nominal

Variable	Type
Previous treatment with cephalosporins	Qualitative, nominal
Previous treatment with carbapenems	Qualitative, nominal
Previous treatment with penicillins	Qualitative, nominal
Previous treatment with proton pump inhibitors	Qualitative, nominal
Previous treatment with antidepressants	Qualitative, nominal
Previous treatment with antihypertensives	Qualitative, nominal
Previous treatment with oral corticosteroids	Qualitative, nominal
Previous treatment with chemotherapy	Qualitative, nominal
Previous treatment with conventional immunosuppressants	Qualitative, nominal
Previous treatment with biological immunosuppressants	Qualitative, nominal
Use of enteral nutritional supplements	Qualitative, nominal
Temperature at diagnosis	Quantitative, continuous
White blood cell count at diagnosis	Quantitative, continuous
Neutrophil count at diagnosis	Quantitative, continuous
Albumin at diagnosis	Quantitative, continuous
Creatinine at diagnosis	Quantitative, continuous
Abdominal pain	Qualitative, nominal
Chills/feverish sensation	Qualitative, nominal
Number of bowel movements	Quantitative, discrete
Stool characteristics	Qualitative, nominal
Ileus during the episode	Qualitative, nominal
Megacolon during the episode	Qualitative, nominal
Intestinal perforation	Qualitative, nominal
Colonoscopy performed	Qualitative, nominal
Pseudomembranous colitis findings on colonoscopy	Qualitative, nominal
Episode severity according to IDSA	Qualitative, ordinal
Zar score	Quantitative, continuous
GEIH-CDI score	Quantitative, continuous
ATLAS score	Quantitative, continuous
C. difficile GDH antigen by IC	Qualitative, nominal
C. difficile toxin antigen by IC	Qualitative, nominal
C. difficile toxin B PCR	Qualitative, nominal
Ct value of *C. difficile* toxin B PCR	Quantitative, continuous
Treatment with metronidazole	Qualitative, nominal
Treatment with vancomycin	Qualitative, nominal
Treatment with fidaxomicin	Qualitative, nominal
Treatment with bezlotoxumab	Qualitative, nominal
ICU admission	Qualitative, nominal
Length of hospital stay (days)	Quantitative, discrete
Cure	Qualitative, nominal
Diarrhea on day 5 of treatment	Qualitative, nominal

Variable	Type
Subsequent recurrence	Qualitative, nominal
Death	Qualitative, nominal

AIDS, acquired immunodeficiency syndrome; COPD, chronic obstructive pulmonary disease; GEIH, *grupo español de infección hospitalaria*; HIV, human immunodeficiency virus; IDSA, Infectious Disease Society of America; PCR, polymerase chain reaction.

Table S2. Concentrations of metabolites in stool samples

Class	Metabolite	Range	Median	IQR	Detection frequency (%)
AA	Histidine	23.003–304.242	60.886	56.012	100
AA	Hydroxyproline	0.46–96.652	4.437	8.906	100
AA	Arginine	5.519–245.408	47.917	59.703	100
AA	Asparagine	0.528–356.585	18.292	39.052	52
AA	Glutamine	2.803–311.306	69.024	113.827	100
AA	Taurine	2.16–1058.786	146.647	381.785	79
AA	Serine	6.515–1334.222	175.481	284.554	100
AA	Ethanolamine	0.3–222.251	38.747	46.286	100
AA	Glycine	24.273–2452.513	748.915	710.244	100
AA	Aspartic acid	5.254–769.754	82.487	132.654	100
AA	Citrulline	1.829–773.472	46.407	143.318	100
AA	Glutamic acid	27.772–6183.135	719.600	1069.554	100
AA	Cystine	4.829–444.088	90.407	126.600	100
AA	Threonine	5.809–875.543	165.269	242.932	100
AA	Hydroxylysine	0.822–53.52	2.788	1.518	100
AA	GABA	5.624–11368.166	163.540	756.371	90
AA	Alanine	58.979–5483.392	1430.027	1379.604	100
AA	Aminoadipic acid	0.429–51.086	1.175	1.269	100
AA	Ornithine	2.151–798.791	151.184	222.969	94
AA	Proline	1.869–1404.74	224.340	395.491	100
AA	Cystathionine	0.919–9.96	2.308	1.303	100
AA	L-Lysine	52.31–2081.267	505.788	727.843	100
AA	Alpha-aminobutyric acid	0.269–311.13	71.462	102.576	94
AA	Tyrosine	4.874–591.939	77.176	187.362	100
AA	Methionine	6.98–342.799	100.486	112.436	100
AA	Phenylalanine	31.269–1393.053	265.619	373.809	100
AA	Tryptophan	39.891–401.46	133.754	124.113	100
BCAA	Valine	29.921–2690.563	533.453	631.101	100
BCAA	Leucine	11.694–1807.896	379.552	370.677	100
BCAA	Isoleucine	130.21–600.28	253.025	145.038	100
SCFA	Acetic acid	0.002–1.15	0.274	0.371	100
SCFA	Propionic acid	0.0–0.009	0.001	0.003	100
SCFA	Isobutyric acid	1.229–538.646	59.426	125.302	100
SCFA	Butyric acid	0.0–0.001	0.000	0.000	94

SCFA	2-Methylbutyric acid	0.0–0.004	0.000	0.001	73
SCFA	Isovaleric acid	0.0–0.013	0.004	0.004	83
SCFA	Valeric acid	0.0–0.002	0.000	0.001	98
SCFA	Heptanoic acid	0.0–0.0	0.000	0.000	100
BA	Lithocholic acid (LCA)	27.32–43069.48	620.160	3802.160	98
BA	Muricholic acid (MuroCA)	8.0–620.72	29.520	41.740	25
BA	Chenodeoxycholic acid (CDCA)	7.92–267639.08	1399.420	9878.690	96
BA	Deoxycholic acid (DCA)	2.76–194450.04	688.760	6133.920	90
BA	Ursodeoxycholic acid (UDCA)	27.56–240902.48	1283.320	6759.000	92
BA	Hyodeoxycholic acid (HDCA)	23.68–4343.52	143.920	588.040	46
BA	Dehydrocholic acid (DHCA)	187.04–34244.28	1351.340	3164.150	50
BA	Cholic acid (CA)	29.96–162533.64	4404.040	18877.580	92
BA	Omega-muricholic acid (wMCA)	19.84–72.64	34.600	20.520	19
BA	Alpha-muricholic acid (aMCA)	0.64–6787.04	76.640	306.820	42
BA	Beta-muricholic acid (bMCA)	119.52–5471.92	842.620	212.140	100
BA	Hyocholic acid (HCA)	0.68–1324.44	149.480	183.360	71
BA	Glycolithocholic acid (GLCA)	0.04–46.28	0.580	1.990	88
BA	Glycochenodeoxycholic acid (GCDCA)	24.0–18320.72	76.220	465.030	100
BA	Glycodeoxycholic acid (GDCA)	0.24–1723.28	33.400	127.970	75
BA	Glycoursodeoxycholic acid (GUDCA)	0.08–969.24	31.700	100.840	100
BA	Glycohyodeoxycholic acid (GHDCA)	0.2–551.8	1.640	8.800	69
BA	Glycodehydrocholic acid (GDHCA)	0.04–3.2	0.320	0.840	40
BA	Glycocholic acid (GCA)	20.6–237028.12	216.360	738.480	100
BA	Glycohyocholic acid (GHCA)	0.04–453.48	1.960	5.990	58
BA	Taurolithocholic acid (TLCA)	0.04–25.16	0.520	2.260	77
BA	Taurochenodeoxycholic acid (TCDCA)	0.04–12814.6	22.700	144.060	92
BA	Taurodeoxycholic acid (TDCA)	0.16–530.92	7.560	23.280	52
BA	Tauroursodeoxycholic acid (TUDCA)	0.04–52.36	1.920	11.030	79
BA	Taurohyodeoxycholic acid (THDCA)	0.04–48.28	1.400	10.550	75
BA	Taurodehydrocholic acid (TDHCA)	0.04–2.6	0.080	0.520	31
BA	Taurocholic acid (TCA)	0.04–13698.96	42.440	281.440	94
BA	Tauro-omega/beta-muricholic acid (Tw/bMCA)	6.32–829.24	16.960	11.830	100
BA	Tauro-alpha-muricholic acid (TaMCA)	1.64–5.08	2.840	2.480	15
BA	Taurohyocholic acid (THCA)	0.12–178.24	2.440	7.810	33
BA	Cholic acid sulfate (CA-S)	0.6–1091.64	74.020	409.690	100
BA	Chenodeoxycholic acid sulfate (CDCA-S)	0.96–5708.04	131.020	580.370	100
BA	Deoxycholic acid sulfate (DCA-S3)	0.52–34462.72	20.240	281.720	98

BA	Deoxycholic acid sulfate (DCA-s2)	0.88–10603.36	241.400	1548.210	100
BA	Glycocholic acid sulfate (GCA-S)	0.72–256.8	8.860	125.760	38
BA	Glycochenodeoxycholic acid sulfate (GCDCA-S7)	0.04–660.28	8.980	18.780	71
BA	Glycodeoxycholic acid sulfate (GDCA-S3)	12.52–1496.6	141.920	241.820	54
BA	Glycolithocholic acid sulfate (GLCA-S3)	0.2–350.32	5.640	40.310	54

AA, amino acids; BA, bile acids; BCAA, branched chain amino acids; SCFA, short chain fatty acids.
